# From Affinity to Proximity Techniques to Investigate Protein Complexes in Plants

**DOI:** 10.3390/ijms22137101

**Published:** 2021-07-01

**Authors:** Sandra M. Kerbler, Roberto Natale, Alisdair R. Fernie, Youjun Zhang

**Affiliations:** 1 Theodor-Echtermeyer-Weg 1, Leibniz-Institut für Gemüse- und Zierpflanzenbau, 14979 Groβbeeren, Germany; kerbler@igzev.de; 2 Max-Planck-Institut für Molekulare Pflanzenphysiologie, Am Mühlenberg 1, 14476 Potsdam-Golm, Germany; roberto.natale@unina.it (R.N.); Fernie@mpimp-golm.mpg.de (A.R.F.); 3 Department of Agricultural Sciences, University of Naples Federico II, 80055 Portici, Italy; 4 Center of Plant Systems Biology and Biotechnology, 4000 Plovdiv, Bulgaria

**Keywords:** affinity purification, proximity labeling, plant protein complex, protein-protein interactions

## Abstract

The study of protein–protein interactions (PPIs) is fundamental in understanding the unique role of proteins within cells and their contribution to complex biological systems. While the toolkit to study PPIs has grown immensely in mammalian and unicellular eukaryote systems over recent years, application of these techniques in plants remains under-utilized. Affinity purification coupled to mass spectrometry (AP-MS) and proximity labeling coupled to mass spectrometry (PL-MS) are two powerful techniques that have significantly enhanced our understanding of PPIs. Relying on the specific binding properties of a protein to an immobilized ligand, AP is a fast, sensitive and targeted approach used to detect interactions between bait (protein of interest) and prey (interacting partners) under near-physiological conditions. Similarly, PL, which utilizes the close proximity of proteins to identify potential interacting partners, has the ability to detect transient or hydrophobic interactions under native conditions. Combined, these techniques have the potential to reveal an unprecedented spatial and temporal protein interaction network that better understands biological processes relevant to many fields of interest. In this review, we summarize the advantages and disadvantages of two increasingly common PPI determination techniques: AP-MS and PL-MS and discuss their important application to plant systems.

## 1. Introduction

The study of biomolecular complexes is crucial in understanding the molecular mechanisms underpinning biological processes, protein function and subcellular protein localization [1,2,3,4]. Biomolecular complexes are principally formed by proteins interacting with other proteins (protein–protein interactions, PPIs), however complexes can also arise through the interaction of proteins with ligands such as nucleic acids, sugars, lipids and hormones [2,3,4]. As the biological function of a protein is defined by its interactions in the cell, an important step in investigating, disrupting or modulating biological processes lies in understanding how and why PPIs occur [1,4]. Advantages of protein complex formation are myriad, starting from greater proximity between substrate and catalyst to enhanced efficiency of whole biochemical pathways.

The field of proteomics has witnessed the development of many innovative methods for the identification and characterization of PPIs [1,3,4]. As method preferences to study protein complexes have changed over time, so too have the possibilities to obtain annotated or predicted protein complexes and composition. Over recent years, proteome-wide studies and computational approaches both point toward a scenario with an increasing number of heteromeric protein complexes being identified [5,6]. The methodology used to predict or identify protein complexes can be categorized in two ways: experimental and computational. Computational or in silico approaches are used to predict PPIs via computer simulations and are dependent on the algorithm used [7]. These predictions are based on high throughput proteomics data (binary or mass spectrometry-based methods), primary structure, 3D structure, domain, evolutionary relationship, genomic methods or a combination of these methods [7,8,9,10]. Experimental approaches are either performed in vitro or in vivo. While in vitro studies are generally performed on a low throughput scale, in vivo studies can be carried out in a high throughput manner. The most common methods used in the study of PPIs are biochemical protein purification or separation (2D gel electrophoresis, 2-DE [11]; blue native polyacrylamide gel electrophoresis, BN-PAGE; size exclusion chromatography, SEC) followed by mass spectrometry (MS), genetic engineering of cellular systems (yeast two hybrid (Y2H) assays and their variants; phage display), arrays (protein arrays or peptides microarrays), structural studies (NMR spectrometry, X-ray crystallography, cryoelectron microscopy) or fluorescence imaging (fluorescence resonance energy transfer, FRET; bimolecular fluorescence complementation BiFC) [1,3,4,12].

Recent studies highlight significant progress in the use of affinity purification and proximity labeling approaches combined with MS-based quantitative proteomics in studying PPIs [5,13,14,15]. Affinity purification mass spectrometry (AP-MS) is a fast, sensitive and targeted approach used to detect interactions between bait (protein of interest) and prey (interacting partners) under near-physiological conditions [16]. This method can be applied to large-scale studies and has been demonstrated to have high intra-and inter-laboratory reproducibility [17]. Similarly, proximity-dependent labeling methods are being increasingly used to detect transient PPIs under native conditions in living cells [14]. As the name suggests, proximity labeling (PL) relies on the principle that proteins must be physically close in order for them to interact and is predicted to be more precise in determining interacting partners [18].

Both AP-MS and PL-MS are powerful techniques that have significantly enhanced our understanding of PPIs. While these methods have become increasingly popular in animal systems, application of these techniques in plants remain underutilized. Combined, AP-MS and PL-MS have the potential to reveal an unprecedented spatial and temporal protein interaction network that better understands biological processes relevant to many fields of interest. For example, AP-MS can be theoretically used to detect transient PPIs as well as interactions involving potentially insoluble proteins such as membrane-associated proteins. Furthermore, PL-MS has the potential to detect hydrophobic interactions under native conditions and has been recently used to investigate membrane contact sites between the endoplasmic reticulum and mitochondria in plants [19]. In this review, we summarize two increasingly common PPI determination techniques: AP-MS and PL-MS and discuss their important application to plant systems.

## 2. Affinity Purification Mass Spectrometry in Plants

Similar to immunopurification or immunoprecipitation (IP), AP utilizes antibodies which can be targeted to the bait, or to a standardized fusion moiety often referred to as an epitope tag [6]. Using protein-specific antibodies, AP-MS has the theoretical advantage of capturing protein complexes under native conditions from plant lysates [5]. However, with limited availability of plant protein antibodies, different bait isoforms that can occlude antibody interaction sites and differing specificities of antibodies, the ability to obtain reliable protein interaction networks remains challenging [4,6]. Therefore, fusion of the bait to various affinity tags has greatly increased the efficacy of this method. Once the bait protein interacts with its respective prey, the resulting complex can be purified from the cell lysate using a matrix that specifically recognizes the affinity tag. Both stable protein complexes and weak PPIs between bait and prey have been detected by AP-MS [20,21]. A critical aspect of this technique lies in protein separation, purification and digestion to reduce the presence of contaminants. Specific protein antibodies can be used to immunoprecipitate the protein of interest under native conditions; however, this approach has only been successfully demonstrated by a few laboratories [5]. While several affinity tags have been developed to allow co-precipitation of prey and bait proteins under native conditions (Table 1), the use of such tags comes with its challenges. Introduction of an epitope tag can result in non-native folding of the tagged protein or steric hindrance of interactions. As bait fused affinity tags generally need to be overexpressed, such expression can influence the physiological properties of the bait or stoichiometry of the complex. Epitope tags can also result in incorrect localization or alternative localization of the protein of interest. It has been shown that overexpression of the bait may result in false positive interactions [6,22]. For these reasons, it is highly recommended that researchers confirm that the chosen epitope tag does not interfere with the endogenous function, localization, or properties of the bait by complementation of the mutant plant line [3,6]. However, these recommendations are not widely utilized due to the time-consuming nature of producing stable transgenic lines and cannot be followed if wild-type plants are used. The use of clustered regularly interspaced short palindromic repeats (CRISPR) technology could help to improve these limitations. Such technology provides researchers with the ability to directly insert affinity tags into endogenous loci without changing the genomic context of the gene and also maintain the native environment to which protein interactions can then be characterized [3,23].

Given the increased sensitivity of MS and the application of novel bioinformatic approaches for accurate data analysis, affinity-based methods have improved considerably in recent years [5,29]. While single tag AP-MS is now widely used in large scale studies, selection of the epitope tag and positioning of the tag at either the N- or C-terminus of protein remains critical. In addition to being an efficient purification handle, some affinity tags also provide benefits such as information regarding subcellar localization of the PPI. For example, fluorescent tags (i.e., green fluorescence protein (GFP), yellow fluorescence protein (YFP) and the mFruits family of monomeric red fluorescent proteins (mRFPs)) allow for localization studies to be performed in parallel to AP-MS studies. The ability to simultaneously monitor both protein localization and expression is useful in investigating whether the recombinant protein occurs under native conditions and if the preyed interactions are biologically relevant. For example, differences in the metabolic roles of glycolytic and TCA cycle enzymes fused with C-terminal GFP were observed in the cytosol and mitochondria respectively [30,31]. In addition, one benefit of using epitope tags is that several proteins can be fused with the same epitope and purified with same method. As a result, background contamination should be consistent across all purifications and should enable the use of the same negative controls, including tag-only constructs or wild-type plants. As shown in Table 1, several types of epitope tags have been successfully applied to AP-MS in plants.

**Figure 1:**
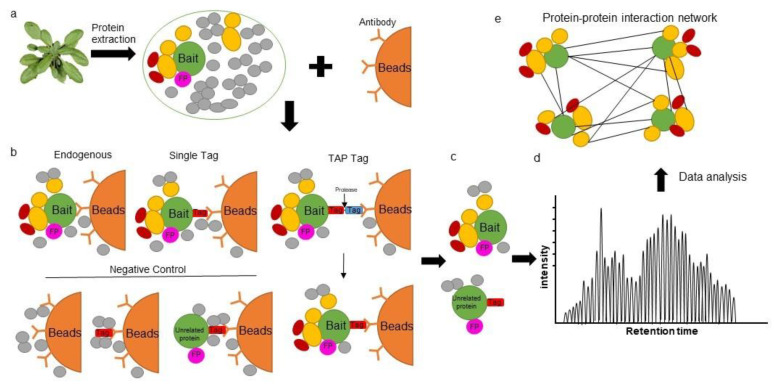
Overview of affinity purification strategies. (**a**) Total protein extraction for affinity purification. (**b**) Bait specific antibodies are linked to beads for protein complex immunoprecipitation under native conditions. Such beads can be used to detect endogenous proteins within a plant, proteins fused displaying a single tag (single affinity purification) or proteins expressing a double (TAP) tag (double affinity purification). Suggested controls used to reduce background contaminants and thus the identification of false positives include using a wild-type plant extract, purification from cells expressing the tag only, or unrelated proteins fused with a tag. (**c**) Several washing steps are used to reduce non-specific interactions. (**d**) Proteins are measured by LC-MS. (**e**) Data analysis to determine a protein–protein interaction network. FP: false positive; UP: unrelated protein.

The main disadvantage of AP-MS however, remains in the ability to fully characterize affinity matrix/epitope tag interaction properties. The identification of non-specific bound proteins is one of the main disadvantages of a single-step purification approach and contaminant proteins associated with either the solid-phase or the epitope tag are hard to distinguish from positive interactors. Thus, the use of proper negative controls such as protein extracts from wild-type plants, mutant lines, or tag-only expressing plants is critical (Figure 1). In principle, unspecific proteins identified in these controls can be simply subtracted from the list of interactors that are identified by the bait. However, given the limitations of AP enrichment and liquid chromatography–mass spectrometry (LC–MS), false positives are still likely. Alternatively, various algorithms can be applied. For example, the SAINT algorithm [32] allows researchers to determine fold change abundance (FC-A), which can be used to filter out potential false positives. Possible interactions can also be evaluated based on the ratio of spectral counts of the bait versus overexpression of an unrelated protein or tag-only controls [33]. Moreover, a second purification step can be introduced to reduce the amount of non-specific binding proteins [5,21]. In tandem affinity purification (TAP), two types of affinity tags linked by a protease cleavage site are fused to a bait protein and expressed in plants. Two affinity purification steps are then performed to obtain reliable interacting partners (Figure 1b). Interestingly, an *Arabidopsis* plant cell culture system has been developed for TAP technology which allows for the high-throughput identification of protein complexes, even with very low sample volumes (25 mg total protein) [5]. GS tags and their derivatives are the most frequently and successfully used TAP tags in plant research [5,34]. A GS tag consists of two immunoglobulin domains of a streptavidin-binding peptide and protein G linked by a unique cleavage site that is recognized by the etch virus protease from tobacco (*Nicotiana tabacum*). Following an initial affinity purification step with immunoglobulin G agarose beads, protein complexes can be incubated with the tobacco etch virus protease to release the complex from the matrix. In a subsequent purification step, the bait protein complex associates with a streptavidin-conjugated bead trap. Following several washing steps, the protein complex is eluted and determined by LC-MS (Figure 1b; [5,21]). In addition, a multifunctional TAP tag (GS^yellow^) has been developed that combines the fluorescent properties of citrine YFP with a streptavidin-binding peptide tag. This double affinity tag can not only be used to determine the subcellular localization of proteins in vivo but also the potential function of the protein through AP [26].

The strength of AP-MS is that it can be used to study PPIs in their relevant plant growth and development biological contexts. For example, studies on specific plant organs including leaves [35], flowers [36] and roots [37], have provided improved information on protein complex organization. Furthermore, AP-MS has the potential to provide insight into posttranslational modification of proteins that may regulate the establishment of spatially or temporally dependent protein interactions [38]. For example, interactions between TCA cycle enzymes and phosphatases have been found using AP-MS in *Arabidopsis* plant cell cultures [31,39,40]. Several posttranslational modification candidates have also been found using AP-MS of glycolytic enzymes in our recent research [30]. These modifications can be directly detected using MS/MS; however, only if they are relatively abundant and if such modifications can withstand the numerous processes involved in protein extraction, purification and MS and MS/MS analyses [41]. Furthermore, given that AP-MS is based on the association of stable complexes, the combination of AP-MS with cross linking has been suggested to greatly improve detection of transient and weak PPIs that are normally lost during protein affinity purification steps [12,22,42].

Chemical cross-linking is a classical approach which is used to freeze PPIs in their native form and has been shown to be especially useful for capturing transient and weak PPIs. For example, membrane protein interactions have been detected in vivo by cross linking with formaldehyde [43]. In the two steps of formaldehyde crosslinking, formaldehyde reacts with a relatively strong nucleophile, most commonly a lysine-amino group from a protein to form a methylol intermediate. Sequentially, the methylol intermediate reacts with another nucleophile, possibly an amino group of a DNA base, to generate a crosslinked product. Thus, formaldehyde could be injected or incubated with plant materials to quickly generate crosslinked protein complexes. Other commonly used cross-linkers include the reversible dithiobis (succinimidyl propionate) during sample extraction to enhance affinity purification of transient and unstable interactions [44]. In addition, a quantitative dimension to AP-MS experiments (q-AP-MS) has been used to overcome issues of non-specific binding of proteins and allows investigation of regulative PPIs under changing physiological conditions [45]. While AP-MS provides a snapshot of the interacting compositions in a multi-subunit complex, it alone, cannot provide insight into the dynamic changes and associations of protein complexes [4].

Two analytical strategies that can be applied to detect dynamic associations of protein complex partners include label-free quantification (LFQ) and stable isotopic labeling. Stable isotope labeling combined with AP-MS has been successfully used to follow the temporal dynamics of PPIs throughout the cell cycle [46] and to investigate protein complexes influenced by different types of cellular perturbation in human and yeast research [47,48]. In *Arabidopsis*, stable isotope labeling combined AP-MS has been used to quantitatively investigate the B-box protein complex, involved in integrating light and hormone signaling pathways during photomorphogenesis from non-specific background proteins [49]. Due to the high cost of labeled substrates and limited labeling efficiency, isotope labeling approaches are restricted in plant research even though it is very sensitive and more accurate than LFQ [50]. In contrast, LFQ technology is easy to perform, cost-effective, and suitable for comparative analyses of large amount samples [51]. LFQ-based technologies use statistical algorithms to analyze relative LC-MS peptide peak abundances based on intensity or counting strategies in multiple replicates [52], so allowing the comparison of samples run at different times. Given that MaxQuant software is an integrated suite of algorithms for the analysis of high-resolution quantitative MS data, its MaxLFQ module is widely used to calculate highly reliable relative LFQ intensity profiles [53] by first searching against the Araport11 database (www.araport.org, accessed on 28 April 2021). Assuming that the enrichment of most proteins (including non-specific background proteins) is kept constant by the design of the experiment, the algorithm promotes the investigation of proteins that are differentially enriched under the tested conditions [54]. Moreover, AP-MS combined with LFQ has also been suggested to assess PPI dynamics during cellular signaling or after cellular perturbations. Given that both tagged bait samples and negative controls can be purified under different conditions or treatments, comparison of quantitative interaction networks could provide the means to assess dynamic protein complex associations. For example, using quantitative (q) TAP in growing maize (*Zea mays*) leaves, growth-regulating factors have been shown to interact with Angustifolia 3 in the division zone, while this interaction was significantly lower in the expansion zone of the same leaves [55]. Another example is the well-characterized strigolactone-dependent interaction (between the receptor protein Dwarf 14 and Suppressor of More Axillary Growth-Like 7), which displays dynamic changes in protein complex composition in response to the hormone [29].

A high-performance affinity enrichment approach for mass spectrometry (AE-MS)- is a technique that combines AP-MS and LFQ and has become an effective method to determine positive PPIs from false positive interactions [56]. Instead of multiple steps of purifying complexes, AE-MS takes advantage of the specific enrichment of interactors in the context of a large number of unspecific background binders by performing a single-step affinity enrichment of endogenously expressed tagged proteins followed by single-run, intensity-based label-free quantitative LC-MS/MS analysis. Although high amounts of non-specific binding proteins are used in the postprocessing pipeline for more accurate normalization and quality control, bait-interacting proteins are expected to be enriched in extracts when compared to negative controls. Given that similar amounts of contaminants are detected under similar conditions in both samples and negative controls, it is easy to eliminate non-specific binding proteins by observing the ratio of interactors versus noise. False positives can also be removed by background normalization, untagged samples and the intensity profiles across all samples. While AE-MS normally requires a minimum of three replicates, this technique has been widely used for large-scale studies as it provides sufficient amounts of data for statistical analyses [57,58]. Both random sample preparation and negative controls are important to determine reliable PPIs networks. To date, AE-MS has been successfully used to characterize several plant PPIs such as dynamin-related proteins interacting with PIN-Formed auxin efflux carriers [59], the protein interaction network of the plant TCA cycle [31,40], MADS domain transcription factor complexes during *Arabidopsis* flower development [57], vascular development-regulating basic helix–loop–helix transcription factor dimers [60] and a glycolysis interaction network [30].

## 3. The Proximity Labeling Method

**Figure 2:**
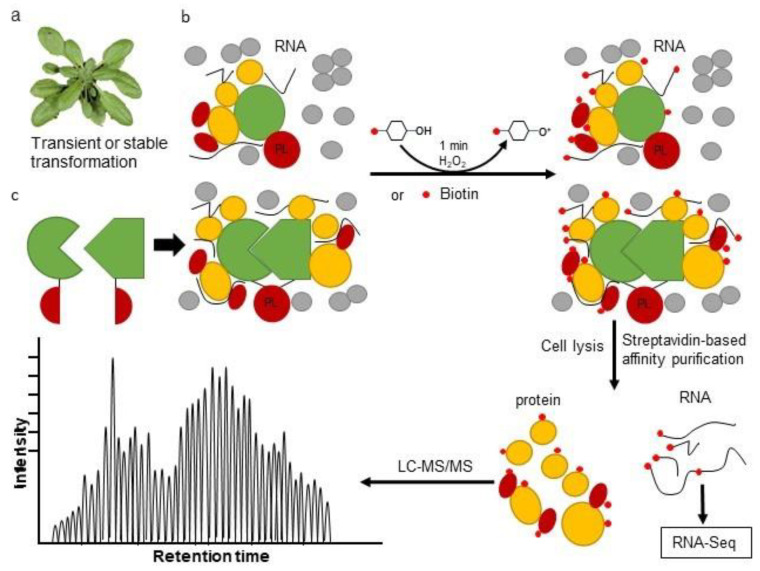
Overview of proximity labeling system. (**a**) Transient and stable protein with proximity-labeling (PL) enzyme transformation. (**b**) PL assay based on the tagged PL enzyme. A biotin ligase or APEX PL enzyme is fused to the target protein and expressed in plants. Upon the addition of a substrate, such as biotin or biotin-phenol and hydrogen peroxide (H_2_O_2_), proteins or RNAs are tagged by biotin. (**c**) Interacting pairs are fused to the PL enzyme at either the N- and C-terminus to investigate the composition of protein complexes. As two proteins interact in cells, the two halves of a split-PL are reorganized as a full PL enzyme and initiate the labeling of proximal partners of the protein complex. After protein extraction and incubating with streptavidin beads, biotin-labeled proteins or RNAs can be enriched for subsequent LC-MS/MS or high-throughput sequencing analysis.

PL-MS is a high-throughput approach for the systematic analysis of PPIs in vivo. While PL-MS is already firmly established in mammalian and unicellular eukaryote systems, application of this technique in planta remains challenging. PL utilizes enzymes that produce reactive molecules that covalently interact with proteins in close proximity. Labeled proteins can be isolated using conventional affinity purification methods and identified via immunoblot analysis or by protein mass spectrometry, Proximity labeling overcomes some of the limitations of AP-MS and Y2H, as abundant soluble proteins as well as insoluble membrane proteins can be effectively enriched under stringent denaturing conditions, which in turn, facilitates their identification. PL can detect weak, transient or hydrophobic PPIs in their native state and provides an unedited spatial and temporal protein interaction network for better understanding of a specific biological process. In addition, fusion of PL enzymes to a minimal targeting motif that restricts proteins to a particular subcellular location or structure, can be used to map the protein population therein [61]. While application of PL-MS to plant systems remains in its infancy, we summarize the recent development of this technology and highlight its potential in studying plant PPIs (Figure 2).

PL-MS has emerged as a powerful tool to characterize PPIs. Over recent years, this technology has grown immensely with the development of new PL enzymes and the application of PL in studying protein interaction networks (including protein-DNA and protein-RNA interactions). Currently, enzyme-mediated activation of radical sources (EMARS), engineered ascorbate peroxidase (APEX) and proximity-dependent biotin identification (BioID) are three commonly used PL technologies [62]. As BioID and its derivatives are highly specific, non-cytotoxic and reproducible, these approaches are increasingly becoming the PL method of choice. Proximity biotinylation is based on the *Escherichia coli* enzyme, BirA. First reported in 2014, BioID relies on the promiscuous activity of a modified BirA protein (mutation of R118G) that releases highly reactive and short-lived biotinoyl-5′-AMP and can modify proteins within a distance of 10 nm [18]. Due to the covalent biotinylation of prey, biotin-labeled targets are stable following stringent cell lysis treatments associated with protein extraction and affinity purification (for example streptavidin beads) with multiple washing steps. This method can also be combined with mass spectrometry measurements to screen for PPIs or detect biotin-labeled proteins with high spatial resolution in living cells. This method has been successfully used to evaluate physiologically relevant PPI networks [14], especially in the detection of transient associations or low-affinity interactions that arise through posttranslational modifications of proteins and signal transduction. An improved smaller enzyme, BioID2, isolated from *Aquifex aeolicus*, has also been produced with the advantage of maintaining the correct localization of a fused protein and requires less biotin for labeling compared to BioID [63].

Similar to BioID, APEX is a 27 kDa monomeric protein which biotinylates prey near the target protein when supplied with ATP, H_2_O_2_ and biotin-phenol at 37 °C [64]. The advantage of using APEX-fused bait proteins is that the time needed to biotinylate all neighboring proteins rich in tyrosine residues is just 1 min, which is significantly faster than the 18–24 h required for BioID. An improved variant of APEX, named “APEX2”, was also developed with increased catalytic activity to reduce the toxic effects of using H_2_O_2_ and biotin-phenol on living cells [65]. Compared to APEX, BioID has the advantage of using non-toxic biotin as a substrate and so avoids the introduction of oxidative stress conditions to cells or tissues. However, as APEX uses quick labeling times, this method has been shown to have greater success in studying dynamic processes such as cell signaling and transient PPIs. Recently, a new PL enzyme and its truncated mutant, termed TurboID (35 kDa) and miniTurboID (28 kDa) respectively, were developed as directed evolution of BirA [66]. The two new versions of BirA combine the advantages of both BioID and APEX and are able to identify interactions involved in fast, dynamic processes without causing damage to living cells [15,67]. Furthermore, several PL enzymes including HRP, APEX2, BioID and TurboID can be split into two parts, similar to the BiFC system, that then can be reconstituted into a functional entity when brought into close proximity. This system is particularly useful for studying membrane contact sites and additional interacting factors of spatio-temporally defined protein complexes [14].

PL technology overcomes several limitations of traditional interaction detection approaches and has been widely used in different biological contexts to highlight different molecular interactions. Furthermore, PL methods demonstrate its potential in detecting interactions with rapid kinetics [68,69]. Several studies underscore the great potential of the PL technique [61,70,71,72], particularly in the detection of weak or transient interactions as well enzyme-substrate interactions that are often difficult to detect by conventional methods. For example, the mitogen-activated protein kinase (MAPK) signaling pathway is often dynamically involved in various physiological processes under different stress responses. While traditional methods have been limited to simultaneously capturing the substrates of MAPK in different states, Dumont and colleagues used APEX2-based PL to map the interactome of p38α and p38γ MAPKs under both steady and activated conditions and revealed novel substrates of p38 [73]. Regarding the proteomic composition of specific regions of an organelle or membrane-associated proteins, information remains scarce due to a lack of techniques to purify these sub-organellar regions. However, PL methods have been successfully applied to study the composition of several large membrane-associated protein complexes, such as the nuclear pore complex (NPC) [18], G-protein-coupled receptors [68,69] and CaV1.2 voltage-gated calcium channels [74]. Indeed, different labs have independently established TurboID-based PL techniques in plant systems including *Arabidopsis*, tomato root cultures and *N. benthamiana* [15,75,76,77]. Comparing the activity of BioID, BioID2, TurboID and miniTurboID in different plant systems, studies have shown that TurboID displays higher efficiency in biotinylating proximal proteins twhen compared to BioID and BioID2 in planta [75,77]. MiniTurboID has the advantage of minimizing the deleterious effect of the tag fusion on the function of target proteins but still shows reduced labeling efficiency compared to TurboID [66].

The use of PL methods is being increasingly applied to different fields of research. This is due to their accessibility, simplicity, and most importantly, potency in probing transient or weak PPIs as well as membrane bound proteins or proteins of low abundance. When performing PL, the first and perhaps most critical step is to choose the enzyme appropriate for ones needs. Secondly, researchers should make sure that the fusion of the PL enzyme to a bait protein does not interfere with its localization or its functions. Lastly, it is important to include appropriate controls to minimize false positives or false negatives. The emerging developments in PL technology provide an incredible opportunity to profile dynamic interaction networks under different conditions, thus offering a global vision of the entire cellular functions, which will greatly advance our understanding of various biological processes.

## 4. Combining Proximity Labeling and Affinity Purification-Mass Spectrometry

While AP-MS results in the identification of proteins that form stable complexes, PL enables the identification of proteins that are in close proximity to the bait, which results in overlapping yet distinct protein identifications. By integrating AP- and PL-MS data, one has the ability to comprehensively characterize a protein’s molecular context and so several combined AP and PL experiments have been trialed. Enzyme combinations allow for both AP-MS and BioID analysis within a single construct and with almost identical protein purification and mass spectrometry (MS) identification procedures such as FLAG-BirA* tag [78,79], Multiple Approaches Combined (MAC)-tag [16] and Strep-Tactin [27] have now been developed. However, there are limitations in combining these two approaches due to the large size of BirA* and the small affinity purification peptide of a Flag or His tag. This strategy of combining AP and PL has not been used in plants to date; however, the generation of specific antibodies for PL tags may facilitate the combination of these two methods in the future.

## 5. Perspectives and Conclusions

The study of PPIs is a rapidly evolving field. AP-MS and PL-MS possess different specificities that can be used according to the type of interactions studied. Moreover, every method has its own strengths and weaknesses. In the future, it is likely that new enzymes will be developed and current systems such as BioID and PL will be further optimized to enhance the applicability of such methods. For AP based methods, the most relevant improvements will be in the reduction of contaminants through new digestion/purification procedures. Another goal will be the extension of their use to different subcellular environments such as vacuoles or peroxisomes, as well as application of these methods in plant species other than in model species [75,77,80]. Another important aspect lies in regard to data analysis; there are a large number of computational tools available to analyze interaction proteomics data. For example, SAINT (Significance Analysis of INTeractome) is an approach based on spectral counting of protein–protein interactions from label-free quantitative proteomics data in AP-MS experiments [81]. Several bioinformatics methods for MS-based proteomics data analysis are well summarized at Chen et al. [82]. In conclusion, the two methods considered in this review offer a broad possibility to study the different interactions that occur in various organisms, shedding light on the complex mechanisms that underlie all biological processes.

## Figures and Tables

**Table 1:** Affinity tags successfully used to investigate plant protein–protein interactions.

Tag	Sequence/Size	Affinity Resin	Elution Conditions	Reference
TAPi tag	45 kDa	Calmodulin binding peptide with two protein A domain	Protein A/low pH	[22,23]
Streptavidin binding peptide (SBP)	WSHPQFEK	Streptavidin	Desthiobiotin	[23,24]
GS^yellow^	37 kDa	Streptavidin-binding peptide tag with citrine yellow fluorescent protein	Desthiobiotin/pH	[23,25,26]
Fluorescent protein (GFP, YFP)	26.9 kDa	Anti-GFP	pH	[13,23,25,26]
GS^rhino^ tag	21.9 kDa	two IgG-binding domains of protein G and a SBP tag	Streptavidin elution buffer [5]	[5,23,27]
Alternative TAP (TAPa)	26 kDa	2 xIgG-BD with 6 XHis and 9 Xmyc	HR3C cleavage/Imidazole/low pH	[23,28]

## Data Availability

Not applicable.
